# Inactivated Rabies Virus Vectored MERS-Coronavirus Vaccine Induces Protective Immunity in Mice, Camels, and Alpacas

**DOI:** 10.3389/fimmu.2022.823949

**Published:** 2022-01-31

**Authors:** Hang Chi, Yanqun Wang, Entao Li, Xiwen Wang, Hualei Wang, Hongli Jin, Qiuxue Han, Zhenshan Wang, Xinyue Wang, Airu Zhu, Jing Sun, Zhen Zhuang, Lu Zhang, Jingmeiqi Ye, Haijun Wang, Na Feng, Mingda Hu, Yuwei Gao, Jincun Zhao, Yongkun Zhao, Songtao Yang, Xianzhu Xia

**Affiliations:** ^1^Changchun Veterinary Research Institute, Chinese Academy of Agricultural Sciences, Changchun, China; ^2^State Key Laboratory of Respiratory Disease, National Clinical Research Center for Respiratory Disease, Guangzhou Institute of Respiratory Health, The First Affiliated Hospital of Guangzhou Medical University, Guangzhou, China; ^3^Food and Drug Inspection Laboratory, Administration for Drug and Instrument Supervision and Inspection, Beijing, China; ^4^Key Laboratory of Zoonosis Research, Ministry of Education, College of Veterinary Medicine, Jilin University, Changchun, China; ^5^Health and Quarantine Laboratory, Guangzhou Customs Technology Center, Guangzhou, China; ^6^Institute of Education, Tsinghua University, Beijing, China; ^7^Jilin Wild Animal Rescue Breeding Center Committee, Jilin Province Northeast Tiger Garden, Changchun, China; ^8^Sate Key Laboratory of Pathogen and Biosecurity, Beijing Institute of Biotechnology, Beijing, China; ^9^Institute of Infectious Disease, Guangzhou Eighth People’s Hospital of Guangzhou Medical University, Guangzhou, China; ^10^Department of Basic Research, Guangzhou Laboratory, Guangzhou, China

**Keywords:** MERS-CoV, vaccine, rabies virus vector, mice, camel, alpaca

## Abstract

Middle East respiratory syndrome coronavirus (MERS-CoV) is an emergent coronavirus that has caused frequent zoonotic events through camel-to-human spillover. An effective camelid vaccination strategy is probably the best way to reduce human exposure risk. Here, we constructed and evaluated an inactivated rabies virus-vectored MERS-CoV vaccine in mice, camels, and alpacas. Potent antigen-specific antibody and CD8^+^ T-cell responses were generated in mice; moreover, the vaccination reduced viral replication and accelerated virus clearance in MERS-CoV-infected mice. Besides, protective antibody responses against both MERS-CoV and rabies virus were induced in camels and alpacas. Satisfyingly, the immune sera showed broad cross-neutralizing activity against the three main MERS-CoV clades. For further characterization of the antibody response induced in camelids, MERS-CoV-specific variable domains of heavy-chain-only antibody (VHHs) were isolated from immunized alpacas and showed potent prophylactic and therapeutic efficacies in the Ad5-hDPP4-transduced mouse model. These results highlight the inactivated rabies virus-vectored MERS-CoV vaccine as a promising camelid candidate vaccine.

## Introduction

The Middle East respiratory syndrome (MERS) is an acute viral infectious disease caused by a zoonotic coronavirus (MERS-CoV) that was first identified in Saudi Arabia in 2012 (1, 2). From September 2012 to July 31, 2021, the WHO has been officially notified of 2,578 laboratory-confirmed MERS cases in 27 countries from the Middle East, Europe, Asia, and Americas, with 888 associated deaths (mortality rate, 34.44%) (3). The high MERS-CoV infection case fatality rate, large geographic distribution, and of licensed MERS-specific vaccines or therapeutics available have drawn global pandemic concern, and MERS was placed in the WHO R&D Blueprint list of priority diseases (4).

Previous studies have shown that dromedary camels serve as an important reservoir for the maintenance and diversification of the MERS-CoV and are the main source of human infection (5, 6). It has been reported that camelids such as dromedary camels, Bactrian camels, alpacas, and llamas are all susceptible to MERS-CoV infection, and animal-to-animal transmission has been demonstrated in the Camelidae family (7–11). Our previous research has demonstrated that zoonotic MERS-CoV infection is occurring in dromedary-exposed populations in Africa, and the extent of MERS-CoV infections is underestimated (12). An effective camelid inoculation strategy to interrupt the transmission cycle is probably the best way to limit MERS-CoV circulation among camelids and reduce human exposure risk, which highlights the development of safe and effective veterinary vaccines (13, 14). Camelid vaccine candidates targeting MERS-CoV S surface glycoprotein developed by different research groups have been reported, mainly utilizing a viral vector-based vaccine, DNA-based vaccine, or subunit vaccine platform (10, 11, 15–18). However, the limited availability of animal models and diverse MERS-CoV clades strains restricted the accurate evaluation of vaccine candidates. The investment in the development of veterinary vaccines is much lower than that of human vaccines. Consequently, the cost is an important consideration regarding whether it will be widely used. Veterinary vaccines must be safe, potent, and effective, and importantly, they must be economical (19). Among the above vaccine platforms, viral vector-based vaccines present advantages of lower production cost and higher immunogenicity; therefore, they are preferred and broadly used in the development of veterinary vaccines (20–22). Rabies virus has been proven to be a favorable vaccine vector and has been widely used to develop recombinant bivalent vaccines. Compared with live viral vector-based vaccines, inactivated recombinant rabies virus-vectored vaccines lessen the concerns of safety and production scalability (23–29). Based on our previous study, the S1 subunit of MERS-CoV spike protein is an effective target able to elicit antigen-specific humoral and cellular immune responses (30). Moreover, viral vector expressing S1 domain induced a better antibody-mediated neutralizing activity compared with the vector expressing full-length S (31). Hence, in the current study, we developed an inactivated viral vector vaccine against MERS-CoV, based on a consensus MERS-CoV truncated S1 subunit glycoprotein, and investigated the immune efficacy of the candidate vaccine in mice, camels, and alpacas against multiple MERS-CoV clade strains. This study highlights inactivated rabies virus vectored MERS-CoV vaccine as a safe, immunogenic, and efficacious vaccine that warrants further assessment.

## Material and Methods

### Virus, Cell Lines, and Animals

Recombinant RABV strain rSRV9 was generated from the vaccine strain SRV9 as previously described (23). The MERS-CoV strains used in this research, including EMC/2012 (NCBI accession No. NC_019843.3), ChinaGD01 (NCBI accession No. KT006149.2) (32), and camel/Nigeria/NV1657/2016 (NCBI accession No. MG923475.1) (33) strains, were isolated from the clinical sample or rescued from BAC infectious clone. BSR cells (a cloned cell line derived from BHK-21 cells), NA cells (derived from mouse neuroblastoma), Huh-7 cells (a hepatocyte carcinoma cell line originally derived from a liver tumor), and Vero 81 cells (derived from African Green monkey kidney) were all purchased from American Type Culture Collection (ATCC) and grown in Dulbecco’s modified Eagle medium (DMEM) (Gibco, San Diego, CA, USA) supplemented with 10% fetal bovine serum (FBS) (Gibco, San Diego, CA, USA). All RABV strains were passaged in BSR cells and titrated by direct fluorescent antibody assay in NA cells. MERS-CoV was passaged in Vero 81 cells and titrated by plaque assay in the same cell line. Six- to eight-week-old specific pathogen-free female C57BL/6 mice were purchased from the Changchun Institute of Biological Products Co., Ltd. (Changchun, China) or Hunan SJA Laboratory Animal Co., Ltd. (Changsha, China). Camels and alpacas were provided by the Wildlife Rescue and Breeding Centre of Jilin Province (Changchun, China).

### Construction and Rescue of Recombinant RABV Expressing MERS-CoV S1 Protein

The recombinant viral vector pD-SRV9-PM-eGFP carrying the enhanced green fluorescent protein (eGFP) gene between phosphoprotein (P) and matrix protein (M) genes of RABV was constructed as previously described. The exogenous gene expression component PE-PS-BsiWI-PmeI was inserted between the stop codon and the transcription stop signal of RABV P gene. To obtain a consensus MERS-CoV S glycoprotein immunogen able to induce broad immune responses, the consensus sequence of S gene was generated by aligning multiple sequences covering the known MERS-CoV clades obtained from the GenBank sequence database and choosing the most common amino acid at each position (**Supplementary Figure 1**). The consensus S sequence was then codon optimized and synthesized by Sangon Biotech Company (Shanghai, China). Considering exogenous antigen incorporation into rabies viral particles related to membrane proximal external anchor region and the soluble form MERS_S1_ protein lacking a membrane anchor, the MERS_S1_ membrane-anchoring chimera protein gene, which contains the synthetic MERS-CoV S1 gene (amino acid 1–725) fused to the gene of human CD4 transmembrane domain (TM) and RABV G protein cytoplasmic domain (CD), was amplified and subcloned into the enzyme cutting sites *Bsi*WI/*Pme*I to replace eGFP gene, generating the recombinant cDNA clone pD-SRV9-PM-MERS_S1_ (34). The recombinant RABV (rSRV9-MERS_S1_) was rescued according to the procedures previously described (23). Briefly, the full-length clone of pD-SRV9-PM-MERS_S1_ and four helper plasmids expressing the N, P, G, and L genes of SRV9 parent virus were co-transfected into BSR cells using Lipofectamine 3000 Transfection Reagent (Invitrogen, San Diego, CA, USA). The rescued rSRV9-MERS_S1_ was detected using anti-rabies monoclonal globulin conjugated with fluorescein isothiocyanate (FITC) (Fujirebio Diagnostics, Seguin, TX, USA) and mouse anti-MERS-S1 monoclonal antibodies (Sino Biological, Beijing, China).

### Electron Microscopy

Inactivated rSRV9-MERS_S1_ viral culture supernatants (virus titer 6.32 × 10^7^ TCID_50_/ml) were loaded onto the carbon-coated TEM support grid after a 5-min incubation at room temperature (RT) and stained with 1% sodium phosphotungstate for 3 min. After wicking the excess liquid off the grid by filter paper, the grid was then examined by JEOL JEM-1200EXII series Transmission Electron Microscope with acceleration voltages ranging at 80 kV.

### Growth Curve

BSR cells were infected at a multiplicity of infection (MOI) of 0.1 with rSRV9-MERS_S1_ and rSRV9 in DMEM. One hour post-incubation, the inoculum was removed. Cells were then washed three times and cultured with DMEM supplemented with 2% fetal calf serum (FCS). The viral culture supernatants were collected at 24-h intervals from 24 to 120 h and titrated in NA cells by direct fluorescent antibody assay.

### Immunofluorescence

Indirect immunofluorescence assay (IFA) was used to detect S protein expression in rSRV9-MERS_S1_-infected cells. NA cells were plated on coverslips in 35-mm-diameter dishes and infected with rSRV9-MERS_S1_ or rSRV9 at a MOI = 1. At 24 h post-infection, cells were fixed in 4% paraformaldehyde (Solarbio Corporation, Beijing, China) at 4°C for 30 min and blocked with phosphate-buffered saline (PBS) containing 1% bovine serum albumin (BSA) at RT for 1 h. Cells were incubated with rabbit anti-MERS-CoV S protein polyclonal antibody (Sino Biologicals, Beijing, China) and mouse anti-RABV G protein monoclonal antibody (Merck Millipore, Hong Kong, China) 1:100 diluted in PBS containing 1% BSA at 37°C for 1 h. Then the cells were washed 3 times with PBST and stained with 594-conjugated goat anti-rabbit antibody (Abcam, Shanghai, China, 1:500 diluted in PBS containing 1% BSA) and 488-conjugated goat anti-mouse antibody (Abcam, Shanghai, China, 1:1,000 diluted in PBS containing 1% BSA) at 37°C for 1 h. After being washed 3 times, cells were stained with an antifade mounting medium with DAPI (Vector Laboratories, Burlingame, CA, USA). Cells were analyzed with an Axio Scope.A1 microscope (Carl Zeiss MicroImaging GmbH, Göttingen, Germany), and representative images were obtained from the ZEN 2012 system (Carl Zeiss MicroImaging GmbH, Göttingen, Germany).

### Recombinant Virus Proliferation, Inactivation, and Purification

BSR cell monolayers in T225 flasks were infected with rSRV9 or rSRV9-MERS_S1_ at a MOI = 0.1. The culture supernatants containing recombinant viruses were collected 4 days post-infection as the viral stock. The titers of the viral stock were determined in NA cells as previously described (23). Beta-propiolactone (BPL) was added at a ratio of 1:3,000 to inactivate the recombinant virus and incubated for 24 h at 4°C, followed by a 30-min incubation at 37°C for BPL hydrolyzation. Then the viral stocks were verified of complete inactivation by direct fluorescent antibody assay in BSR cells and NA cells. For the virus purification, the inactivated viral stocks were precipitated with 2% zinc acetate solution and purified by ultracentrifugation on a discontinuous sucrose gradient. The purified inactivated virus particles were resuspended with sterile PBS, determined with the BCA Protein Assay Kit (Thermo Fisher, Waltham, MA, USA) for protein concentration.

### Sodium Dodecyl Sulfate–Polyacrylamide Gel Electrophoresis and Western Blotting

For total protein analysis, 10 μg of purified inactivated recombinant virus particles rSRV9 or rSRV9-MERS_S1_ was separated on a 12% sodium dodecyl sulfate (SDS)–polyacrylamide gel and stained with Coomassie blue staining solution (Beyotime, Shanghai, China) according to the manufacturer’s instructions. For Western blotting analysis of target bands, an 12% SDS–polyacrylamide gel was transferred onto a 0.45-μm nitrocellulose blotting membrane (GE Healthcare, Madison, WI, USA) using mouse anti-MERS-S1 monoclonal antibodies (Sino Biologicals, Beijing, China) and mouse anti-RABV-G monoclonal antibodies (Merck Millipore, Hong Kong, China).

### Neurovirulence

For analysis of viral neurovirulence, 4- to 6-week-old Institute of Cancer Research (ICR) adult mice and 14-day-old ICR suckling mice purchased from the Changchun Institute of Biological Products Co., Ltd. (Changchun, China) were respectively administered intracerebrally (i.c.) with injections of 25-μl serial dilutions of the recombinant virus rSRV9-MERS_S1_ and the parental virus rSRV9. Mice were individually weighed periodically and monitored for signs of encephalitis and morbidity for 21 days. Further decreasing the age of suckling mice, 5-day-old ICR mouse pups were injected with rSRV9-MERS_S1_ or rSRV9 and monitored daily for clinical signs of encephalitis for 28 days. The number of surviving mice was recorded daily.

### Mice Immunizations

To determine the appropriate vaccine form of the recombinant rabies virus-vectored MERS-CoV vaccine, groups of 10 six- to eight-week-old specific pathogen-free female C57BL/6 were injected intramuscularly (i.m.) in the quadriceps muscle with 2 × 10^7^ TCID_50_ of the recombinant virus rSRV9-MERS_S1_ on day 0 or the BPL inactivated rSRV9-MERS_S1_ plus Imject Alum adjuvant (Thermo Fisher, Waltham, MA, USA) on day 0 (one-dose group) or days 0 and 14 (two-dose group). Vector control groups were injected with the same dose of the live or inactivated parental virus rSRV9 at the same time points. Meanwhile, the Alum adjuvant control group and mock control group were set up. Blood samples of each group were collected by retro-orbital plexus puncture on days 14, 21, and 28 following the first immunization (day 0).

To evaluate the immunogenicity of the inactivated recombinant rabies virus-vectored MERS-CoV vaccine, 6- to 8-week-old specific pathogen-free female C57BL/6 mice were randomly divided into four groups of 12 mice each. Mice in the experimental group were i.m. injected in the quadriceps muscle with the inactivated recombinant rabies virus-vectored MERS-CoV vaccine (BPL inactivated virus plus equal volumes of Imject Alum adjuvant) on days 0 and 14. Mice in the control group received the inactivated parental rabies virus vaccine or PBS plus Alum adjuvant or PBS at the same time points. At weeks 2, 4, 5, and 6 and months 2, 4, 8, and 10 following the primary immunization (week 0), blood samples were collected by retro-orbital plexus puncture.

### Detection of Antibody Responses

Sera were isolated for the detection of antibody responses. Indirect ELISA measurement of MERS-CoV S-specific IgG in sera was conducted as previously described. Neutralizing antibody titers of sera against MERS-CoV were detected by a pseudovirus-based neutralization assay and live virus neutralization assay as previously described (30, 35). For pseudovirus assay, human MERS-CoV KOR/HIN strain, dromedary camel MERS-CoV D1271 strain, and Human betacoronavirus 2c EMC/2012 strain pseudoviruses were used. For live virus assay, Human betacoronavirus 2c EMC/2012 strain, MERS-CoV isolate ChinaGD01 strain, and dromedary/Nigeria/HKU NV1657 strain respectively from Clade A, B, and C were used to evaluate the cross-neutralizing activity among various MERS-CoV clades. Neutralizing antibody titers of sera against RABV were measured using fluorescent antibody virus neutralization (FAVN) (36).

### Intracellular Cytokine Staining

One and 4 weeks following the second immunization, 3 mice from each group were randomly selected and euthanized. Spleens were harvested into a tissue culture dish and teased apart into single-cell suspensions by pressing through a 5-ml syringe. After ammonium–chloride–potassium (ACK) lysing buffer treatment, splenocytes were washed twice and maintained in Roswell Park Memorial Institute (RPMI) 1640 medium (Gibco, San Diego, CA, USA) enriched with 10% FBS (Gibco, San Diego, CA, USA). Cells were then added to a 6-well plate (2 × 10^6^ per well) and stimulated with 20 μM of peptide S434 (a previously identified MERS-CoV-specific immunodominant CD8^+^ T-cell epitope in C57BL/6 mice (37)) in the presence of protein transport inhibitor cocktail (brefeldin A and monensin) (BD Biosciences, Franklin, VA, USA) for 6 h at 37°C in 5% CO_2_. All cells were then labeled with surface staining antibodies, fixed and permeabilized with the Cytofix/Cytoperm Solution (BD Biosciences, Franklin, VA, USA), and labeled with anti-intracellular cytokine antibodies. All labeled cells were analyzed by a FACSCalibur™ Flow Cytometer (BD Biosciences, Franklin, VA, USA). The following antibodies were purchased from BD Biosciences and used for label cells: PE anti-mouse CD8a (Clone # 53-6.7), PE-Cy7 anti-mouse IFN-γ (Clone # XMG1.2), APC anti-Mouse TNF-α (Clone # MP6-XT22), and PE-Cy7 anti-mouse IL-2 (Clone # JES6-5H4). Analysis of fluorescence-activated cell sorting (FACS) data was performed using the FlowJo flow cytometry analysis software.

### IFN-γ ELISpot Assays

One and 4 weeks following the second immunization, splenocytes from 3 mice of each group were isolated as described above. Then 5 × 10^5^ cells were added to each well of a 96-well ELISpot plate pre-coated with IFN-γ (Mabtech AB, Stockholm, Sweden) and stimulated with or without peptide S434 (10 μM; specific peptide antigen) and concanavalin A (10 μg/ml; positive control). After being incubated at 37°C in 5% CO_2_ for 24 h, cells producing IFN-γ in the splenocytes were measured using mouse enzyme-linked immunospot (ELISpot) kits (Mabtech AB, Stockholm, Sweden) according to the manufacturer’s instructions. Spot-forming cells (SFCs) were enumerated by an automated ELISpot reader (AID ELISPOT reader-iSpot, AID GmbH, Penzberg, Germany).

### ELISA Measurement of Cytokines

Splenocytes were isolated, cultured (1 × 10^6^ cells/ml) in a 6-well plate, stimulated with 10 μM of specific peptide S434 as described above, and then incubated at 37°C in 5% CO_2_. After 48 h, cell-free culture supernatants were harvested. Levels of IFN-γ, TNF-α, and IL-2 were measured using mouse ELISA development kits (Mabtech AB, Stockholm, Sweden) according to the manufacturer’s instructions.

### MERS-CoV Challenge of the Vaccinated Mice

For the live MERS-CoV challenge, 6- to 8-week-old specific pathogen-free female C57BL/6 mice purchased from the Changchun Institute of Biological Products Co., Ltd. (Changchun, China) were randomly divided into two groups. A total of 8 mice were i.m. injected with the inactivated recombinant rabies virus-vectored MERS-CoV vaccine on days 0 and 14, while the other 8 mice received the inactivated parental rabies virus vaccine as a negative control. On day 24, blood samples of each group were collected by retro-orbital plexus puncture. Mice were sensitized to MERS-CoV infection after prior transduction with adenovirus 5 expressing human DPP4 (Ad5-hDPP4) as previously described (37). Mice were transduced with Ad5-hDPP4 5 days before the intranasal challenge with 1 × 10^5^ PFU MERS-CoV (EMC/2012 strain) on day 42. At 3 days post-infection, the lungs of 3 mice of each group were harvested and manually homogenized into 3 ml of PBS. Lung virus titers were determined in Vero 81 cells and expressed as PFU/g of tissue.

### Camel Immunizations

For the immunization experiment of camels, four Bactrian camels (2 females and 2 males) were allocated into three groups. Camels were injected subcutaneously (s.c.) in the neck (distributed in multiple injection sites) with 5 ml of the inactivated recombinant rabies virus-vectored MERS-CoV vaccine (n = 2) or the inactivated parental rabies virus vaccine (n = 1) or PBS (n = 1) two times at 4-week intervals. The blood samples of camels were collected before the first immunization and collected at weeks 4 and 8 after the first immunization by two licensed veterinarians.

### Alpaca Immunizations

For the immunization experiment of alpacas, three alpacas (2 females and 1 male) were allocated into two groups. Alpacas (n = 2) in the experimental group received 3 ml of the inactivated recombinant rabies virus-vectored MERS-CoV vaccine by multiple sites through s.c. injection in the neck two times at 3-week intervals. Alpacas (n = 1) in the control group received the same volumes of PBS at the same time points. The blood samples of alpacas were collected before the first immunization and collected at weeks 3, 6, 12, and 28 after the first immunization by two licensed veterinarians.

### Selection and Expression of Recombinant MERS-CoV-S-Specific VHHs

Alpacas (n = 2) in the experimental group were boosted with the inactivated recombinant rabies virus-vectored MERS-CoV vaccine three times at weeks 43, 46, and 49. On day 7 after the last immunization, blood from the immunized alpacas was collected, and then their peripheral blood mononuclear cells (PBMCs) were isolated using Ficoll-Paque PLUS gradient centrifugation (GE Healthcare, Madison, WI, USA). PBMCs were counted, and then total RNA was extracted using TRIZOL LS reagent (Invitrogen, San Diego, CA, USA). cDNA was synthesized by reverse transcription-PCR using a SuperScript III Reverse Transcriptase kit (Invitrogen, San Diego, CA, USA). Alpaca variable domains of heavy-chain-only antibody (VHH) genes were amplified using a two-step nested PCR approach with HotStarTaq Plus DNA polymerase (Qiagen, Hilden, Germany) using the following primer: the first PCR forward primer VHH-L-F (5′-GGTGGTCCTGGCTGC-3′) and the reverse primer CH2-R (5′-GGTACGTGCTGTTGAACTGTTCC-3′) were used to amplify the N-terminal IgG heavy-chain fragment; the second nested PCR forward primer AlpVh-FR1-NheI (5′-CTAGCTAGCATGGCCCAGKTGCAGCTCGTGGAGTCNGGNGG-3′) and the reverse primers (AlpVHHR1-BamHI, 5′-CGCGGTACCGGGGTCTTCGCTGTGGTGCG-3′, AlpVHHR2-BamHI, 5′-CGCGGTACCTTGTGGTTTTGGTGTCTTGGG-3′) were used to amplify the VHH repertoire (~300 to 450 bp). Amplified VHH DNA was digested with *Nhe*I and *Bam*HI restriction enzymes and cloned into yeast surface display vector pCTCON-2 to construct the alpaca immune VHH library displayed on the surface of yeast *Saccharomyces cerevisiae*. Purified MERS-S trimer was used as antigen bait to select the VHH library. After three rounds of FACS with a FACSAri II cell sorter (BD Biosciences, Franklin, VA, USA), DNA plasmids containing VHH coding sequences were extracted from the sorted antigen-binding yeast population and then transformed into *Escherichia coli* DH5α for sequencing. Distinct VHH sequences were identified among the total sequences analyzed. To extend the *in vivo* half-life through increasing antibody size, the selected VHH genes were cloned into the backbone of antibody expression vectors containing a C-terminal Fc domain of human IgG1. The recombinant VHH-Fcs were expressed in 293T cells by transient transfection and then purified.

### Antibody Treatment and MERS-CoV Infection of Mice

To evaluate the antibody contribution to the protection, the prophylactic and therapeutic efficacies against the MERS-CoV challenge were assessed in Ad5-hDPP4-transduced mice. Briefly, 5 days after intranasally transduction with Ad5-hDPP4, mice were infected intranasally with 1 × 10^5^ PFU MERS-CoV (EMC/2012 strain). Mice in the experimental group were intravenously injected with 200 μl of immune sera (from immunized alpacas and camels) or VHH1-Fc 1 day before or after MERS-CoV infection. Mice in the control group intravenously received the same dose of negative sera from healthy alpacas, camels, or negative control antibody (anti-HIV antibody 2G12) at the same time points. The lungs were harvested at 3 days post-infection and manually homogenized in PBS. Virus titers were determined in Vero 81 cells and expressed as FFU/g of tissue.

## Results

### Generation and Validation of the Recombinant Rabies Virus Expressing MERS-CoV S1 Protein

**Figure 1:**
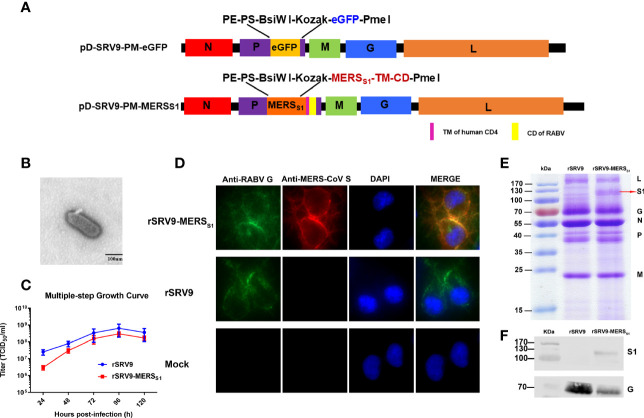
Characterization and validation of rSRV9-MERS_S1_. **(A)** Schematic of the candidate vaccine rSRV9-MERS_S1_. The MERS_S1_ membrane-anchoring chimera protein gene, which contains MERS-CoV S1 gene fused to the gene of human CD4 transmembrane domain (TM) and RABV G protein cytoplasmic domain (CD), was amplified and subcloned into the enzyme cutting sites *Bsi*WI/*Pme*I of the recombinant plasmid containing full-length RABV cDNA (pD-SRV9-PM-eGFP), generating the recombinant cDNA clone pD-SRV9-PM-MERS_S1_. **(B)** TEM detection of rSRV9-MERS_S1_. The samples of inactivated rRABV-MERS_S1_ viral culture supernatants were stained with 1% sodium phosphotungstate. Bar = 100 nm. **(C)** Multiple-step growth curves of rSRV9-MERS_S1_ and rSRV9 on BSR cells (multiplicity of infection (MOI) = 0.1). Cell culture supernatants were then harvested at 24, 48, 72, 96, and 120 h post-infection. Data were obtained using GraphPad Prism version 9.0 (GraphPad software). Data are shown as mean ± SD. **(D)** Validation of the expression of MERS-CoV S1 protein and RABV G protein in rSRV9-MERS_S1_-infected NA cells by indirect immunofluorescence staining. NA cells infected with rSRV9 or mock-infected NA cells were used as controls. **(E)** Sodium dodecyl sulfate–polyacrylamide gel electrophoresis (SDS-PAGE) analysis of viral protein expression in purified rSRV9-MERS_S1_ virions and rSRV9 virions. **(F)** Western blotting detection of MERS-CoV S1 protein and RABV G protein expressions in purified recombinant virus particles rSRV9-MERS_S1_ using mouse anti-MERS-S1 monoclonal antibodies and mouse anti-RABV-G monoclonal antibodies. Purified parental virus particles rSRV9 were used as control.

Recombinant genomic cDNA clone pD-SRV9-PM-MERS_S1_ was constructed based on the previously (23) established rabies virus SRV9 strain reverse genetics system (**Figure 1A**). Recombinant rabies virus (rSRV9-MERS_S1_) was successfully rescued in BSR cells, showing typical bullet-shaped morphology under transmission electron microscopy (**Figure 1B**). Similar to the other recombinant rabies viruses (25, 38), the growth kinetics of recombinant viruses is slower than that of the parental rabies virus. Although the titers of rSRV9-MERS_S1_ were lower than those of rSRV9 at 24 and 48 hpi, which may be related to the expression of MERS-CoV S1, overall, rSRV9-MERS_S1_ showed similar growth kinetics as rSRV9 in BSR cells, with the peak titers reaching 2 × 10^8.5^ TCID_50_/ml (**Figure 1C**). The expression of MERS-CoV S1 and RABV G proteins in rSRV9-MERS_S1_-infected BSR cells was identified by indirect immunofluorescence staining (**Figure 1D**). The neurovirulence of the recombinant virus rSRV9-MERS_S1_ versus the parental virus rSRV9 was also evaluated. Four- to 6-week-old ICR adult mice and 14-day-old ICR suckling mice i.c. injected with serial dilutions of rSRV9-MERS_S1_ did not show any clinical signs or lethality. On the other hand, the results of intracerebral challenge in 5-day-old ICR suckling mice demonstrate that the neurovirulence of rSRV9-MERS_S1_ was reduced compared to that of the parental virus rSRV9 (**Table 1**), indicating an increased safety profile. Similar results were also observed in other rabies-vectored vaccines, and neurovirulence attenuation of rSRV9-MERS_S1_ may be due to its slower growth kinetics than that of the parental rabies virus (25, 38).

**Table 1:** The neurovirulence of rSRV9-MERS_S1_ compared to rSRV9 in 5-day-old suckling mice.

Virus	Dose (TCID_50_)	Survival (%)	Mean (± SD) endpoint (days)
rSRV9	10^1^	20	11.6 ± 1.7
	10^3^	0	9.5 ± 1.6
	10^5^	0	9.3 ± 0.9
rSRV9-MERS_S1_	10^1^	80	12.5 ± 2.1
	10^3^	0	11.5 ± 1.5
	10^5^	0	9.8 ± 1.4

Institute of Cancer Research (ICR) mouse pups aged 5 days were intracerebrally (i.c.) injected with rSRV9-MERS_S1_ or rSRV9 and monitored daily for clinical signs of encephalitis for 28 days.

To confirm the incorporation of MERS-CoV S1 protein into the recombinant rabies virus particles, the inactivated viral stocks were purified by a discontinuous sucrose gradient. As shown by SDS-PAGE analysis, the L (180 kDa), G (65 kDa), N (55 kDa), P (38-41 kDa), and M (180 kDa) proteins of RABV were detectable in both purified rSRV9-MERS_S1_ and rSRV9 virions, while the protein band corresponding in size to the MERS-CoV S1 protein (120 kDa) was only detected in rSRV9-MERS_S1_ purified virions (**Figure 1E**). The incorporation of MERS-CoV S1 protein into the RABV virions was further confirmed by Western blotting analysis (**Figure 1F**). All the results above indicated the successful construction and stable expression of rSRV9-MERS_S1_, which may have the potential to serve as an effective inactivated vaccine candidate.

### Strong Antigen-Specific Antibody Responses Was Induced by the Inactivated Rabies Virus-Vectored MERS-CoV Vaccine in Mice

**Figure 2:**
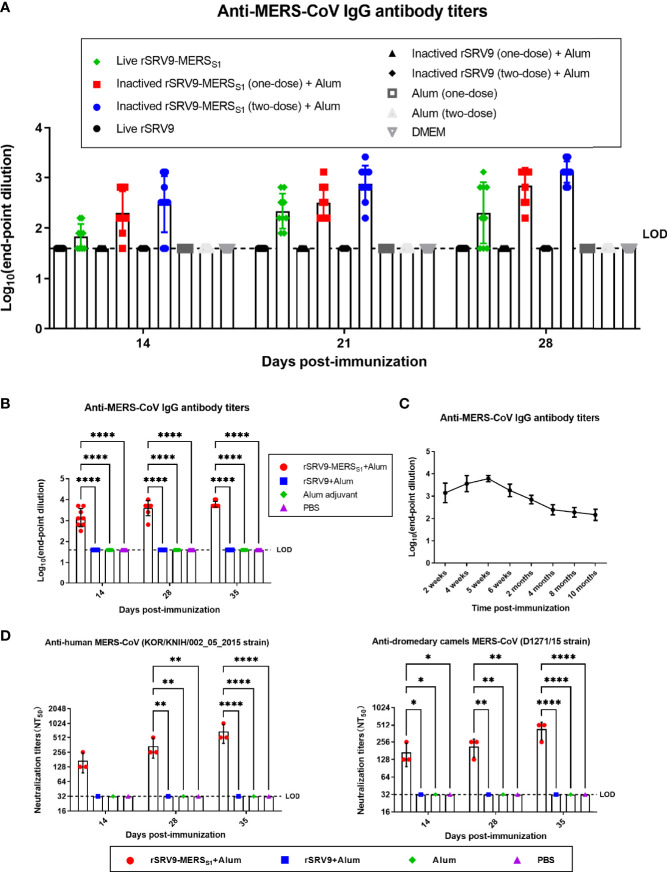
Humoral immune responses against MERS-CoV elicited by the inactivated rabies virus-vectored MERS-CoV vaccine in mice. **(A)** Evaluation of an appropriate vaccine from recombinant rabies virus-vectored MERS-CoV vaccine candidates (n = 9 per group). The immunogenicity of the vaccine candidates with different forms (live and inactivated) and delivery doses (one dose assayed for both live and inactivated vaccines, two doses assayed for inactivated vaccine) was evaluated by anti-MERS-CoV IgG antibody through indirect ELISA. Endpoint dilution titers were calculated at the indicated time points. **(B)** The total anti-MERS-CoV IgG antibody titers of sera from each group of mice (n = 8) were assessed on days 14, 28, and 35 after the first immunization and shown as endpoint dilution titers. **(C)** The dynamic changes and duration of serum antibodies from each group of mice (n = 8) were evaluated by indirect ELISA at the indicated time. **(D)** The cross-neutralizing activity against divergent MERS-CoV isolates (human MERS-CoV KOR/HIN strain and dromedary camel MERS-CoV D1271 strain) of sera from each group of mice (n = 3) were evaluated by a pseudovirus-based neutralization assay. Data are shown as mean ± SD. Data were obtained using GraphPad Prism version 9.0 (GraphPad software). Significance was calculated using a two-way ANOVA with multiple comparisons tests (not indicated in graph; *p < 0.05, **p < 0.01, ****p < 0.0001).

To select an appropriate vaccine form of the recombinant rabies virus-vectored MERS-CoV vaccine candidates, several recombinant rabies virus-based vectored MERS-CoV vaccine candidates were developed based on the rescued rSRV9-MERS_S1_; in detail, the immunogenicity of these vaccine candidates was evaluated in mice with different forms (live and inactivated rabies virus-vectored MERS-CoV vaccine) and delivery doses (one dose assayed for both live and inactivated vaccines, two-dose vaccination assayed for the inactivated vaccine). Of the MERS-CoV vaccine candidates developed, the inactivated rabies virus-based vectored MERS vaccine with two immunizations elicited the most robust antibody response in immunized mice (**Figure 2A**) and thus was selected for further experiments. Anti-MERS-CoV IgG antibody levels were measured by indirect ELISA and shown as endpoint dilution titers. Mice immunized with the inactivated rabies virus-vectored MERS-CoV vaccine induced significant MERS-CoV S protein-specific IgG as compared to the controls, with an increasing trend after each immunization (**Figure 2B**). The dynamic changes and duration of the anti-MERS-CoV antibody were also evaluated. As shown in **Figure 2C**, the total anti-MERS-CoV IgG antibody titers peaked at 5 weeks after the first immunization and were still detectable 10 months after the first immunization. Of note, the sera from inactivated vaccine immunized mice were able to neutralize multiple MERS-CoV pseudoviruses, including human MERS-CoV KOR/HIN strain and dromedary camel MERS-CoV D1271 strain, indicating the cross-neutralizing activity against divergent MERS-CoV isolates from both human and camels (**Figure 2D**). These results demonstrated that the inactivated rabies virus-vectored MERS-CoV vaccine induces a robust binding and neutralizing antibody response in mice.

### Potent Antigen-Specific CD8^+^ T-Cell Responses Were Elicited by the Rabies Virus-Vectored MERS-CoV Vaccine in Mice

**Figure 3:**
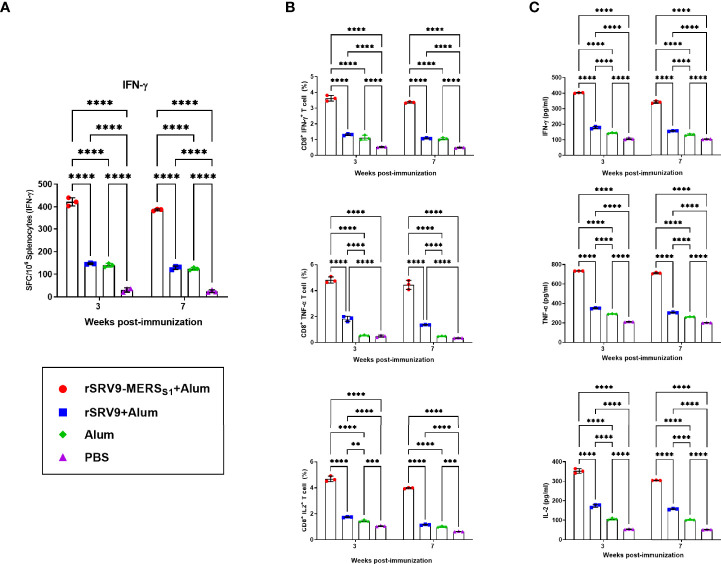
CD8^+^ T-cell responses elicited by the inactivated rabies virus-vectored MERS-CoV vaccine. Splenocytes from each group of mice (n = 3) were harvested 1 and 4 weeks following the second immunization and evaluated for the vaccine-induced specific cellular immune responses. **(A)** The MERS-CoV-specific IFN-γ activities in splenocytes were measured using commercial ELISpot kits. Spot-forming cells (SFCs) secreting IFN-γ were enumerated in an automated ELISpot reader and shown as mean responses in each group at the indicated time points. **(B)** The frequencies of MERS-CoV-specific IFN-γ-, TNF-α-, and IL-2-secreting CD8^+^ T cells in splenocytes were evaluated using intracellular cytokine staining (ICS) assays. **(C)** Levels of IFN-γ, TNF-α, and IL-2 secreted by splenocytes were measured using commercial ELISA kits. Data are shown as mean ± SD. Data were obtained using the GraphPad Prism version 9.0 (GraphPad software). Significance was calculated using a two-way ANOVA with multiple comparisons tests (not indicated in graph; **p < 0.01, ***p < 0.001, ****p < 0.0001).

To monitor the vaccine-induced specific cellular immune responses of both T-cell activation and memory period, splenocytes were harvested 1 and 4 weeks following the second immunization. IFN-γ responses after stimulation with MERS-CoV-specific peptides were measured by mouse enzyme-linked immunospot (ELISpot) assay. As indicated in **Figure 3A**, significantly more IFN-γ-secreting cells were detected in splenocytes from the rabies virus-vectored MERS-CoV vaccine immunized mice than the controls. Furthermore, the frequencies of MERS-CoV-specific IFN-γ-, TNF-α-, and IL-2-secreting CD8^+^ T cells in splenocytes were counted and analyzed using intracellular cytokine staining (ICS) assays. The proportion of CD8^+^ T cells that produce IFN-γ, TNF-α, and IL-2 was superior in the rabies virus-vectored MERS-CoV vaccine immunized mice, indicating the vaccination markedly increased the antigen-specific CD8^+^ T-cell responses in mice (**Figure 3B**). Similar results were observed for the levels of cytokines secreted by splenocytes assayed by ELISA (**Figure 3C**). Notably, despite there being a downswing of the vaccine-induced specific cellular immune responses between T-cell activation and memory period, trends remained consistent among groups in each phase.

### Efficient Protection of MERS-CoV-Infected Ad5-hDPP4-Transduced Mice by the Rabies Virus-Vectored MERS-CoV Vaccine

**Figure 4:**
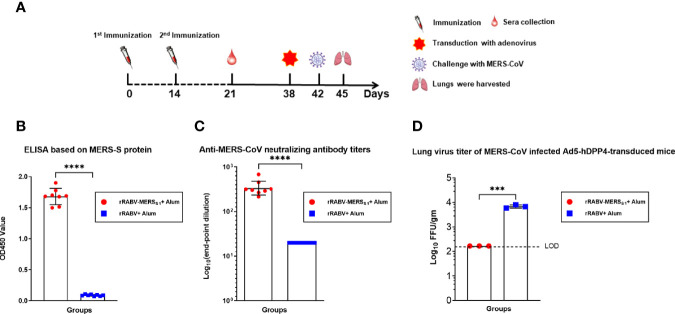
Immunization with the rabies virus-vectored MERS-CoV vaccine provides protection in MERS-CoV-infected Ad5-hDPP4-transduced mice model. **(A)** The schematic of the live challenge experiment. Six- to eight-week-old specific pathogen-free female C57BL/6 mice (n = 8 per group) were intramuscularly (i.m.) injected with either the inactivated recombinant rabies virus MERS-CoV vaccine or inactivated parental rabies virus vaccine on days 0 and 14; sera from each group of mice (n = 8) were collected on day 21. All mice were transduced with Ad5-hDPP4 5 days as previously described before intranasal challenge with 1 × 10^5^ PFU MERS-CoV (EMC/2012 strain) on day 42, and then the lungs from each group of mice (n = 3) were harvested for virus titration. **(B)** Binding and **(C)** neutralizing activities of sera collected on day 21 were respectively measured by indirect ELISA and live MERS-CoV (EMC/2012 strain). **(D)** On day 3 post-infection, virus titers in the lungs were measured on Vero 81 cells and expressed as FFU/g tissue. Data are shown as mean ± SD. Data were obtained using GraphPad Prism version 9.0 (GraphPad software). An unpaired Student’s t-test (two-tailed) was used for statistical analysis, and the relevant p-values are indicated (not indicated in graph; ***p < 0.001, ****p < 0.0001).

The protective immunity of the rabies virus-vectored MERS-CoV vaccine was evaluated in the Ad5-hDPP4-transduced mouse model and determined by virus load in the infected lungs (**Figure 4A**). Immune sera at day 21 from the mice receiving the rabies virus-vectored MERS-CoV vaccine demonstrated binding activity to MERS-S (**Figure 4B**) and neutralizing activities against live MERS-CoV (EMC/2012 strain) (**Figure 4C**). As shown in **Figure 4D**, all the rabies virus-vectored MERS-CoV vaccine immunized mice had no detectable viral loads on day 3 post-infection, while the control mice had on average as high as 10^4^ PFU/g of virus in their lungs, demonstrating that the vaccination reduced viral replication and accelerated virus clearance in the lungs.

### Protective Antibody Responses Elicited by the Rabies Virus-Vectored MERS-CoV Vaccine in Camels

**Figure 5:**
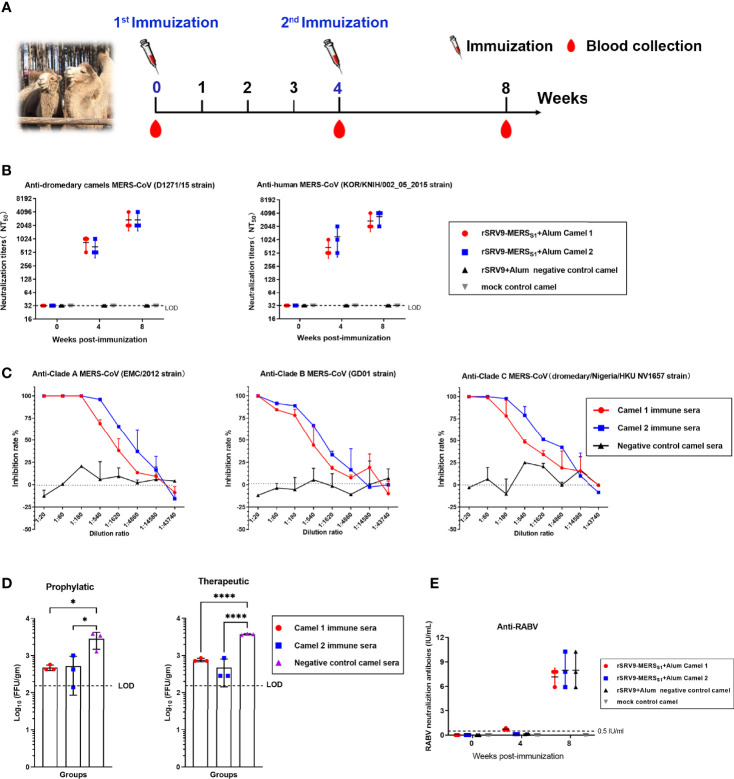
Humoral immune responses elicited by the inactivated rabies virus-vectored MERS-CoV vaccine in camels. **(A)** The schematic of the camel immunization experiment. Four Bactrian camels were injected subcutaneously (s.c.) in the neck with 5 ml of inactivated recombinant rabies virus-vectored MERS-CoV vaccine (n = 2) or the inactivated parental rabies virus vaccine (n = 1) or phosphate-buffered saline (PBS) (n = 1) two times at 4-week intervals. Sera of camels from each group were harvested before the first immunization and collected at weeks 4 and 8 after the first immunization. **(B)** Sera cross-neutralizing activity against representative human and camel MERS-CoV strains (human MERS-CoV KOR/HIN strain and dromedary camel MERS-CoV D1271 strain) were evaluated by a pseudovirus-based neutralization assay. **(C)** The cross-neutralizing activity against live viruses among the known MERS-CoV clade A (EMC/2012 strain), clade B (ChinaGD01 strain), and clade C (dromedary/Nigeria/HKU NV1657 strain) were measured by plaque reduction neutralizing assay. **(D)** Prophylactic and therapeutic efficacies of passive transfer with the rabies virus-vectored MERS-CoV vaccine camel immune sera collected at 8 w.p.i in MERS-CoV-infected Ad5-hDPP4-transduced mice (n = 3 per group). A total of 200 μl of camel immune sera collected was intravenously transferred 1 day before or after MERS-CoV (EMC/2012 strain) infection. Mice in the control group (n = 3) intravenously received the same dose of negative sera from healthy camels at the same time points. **(E)** The RABV-specific neutralizing antibody titers of sera from each group of mice (n = 3) were evaluated using fluorescent antibody virus neutralization (FAVN). Data are shown as mean ± SD. Data were obtained using GraphPad Prism version 9.0 (GraphPad software). Significance was calculated using a one-way ANOVA with multiple comparisons tests (not indicated in graph; *p < 0.05, ****p < 0.0001).

Camels were immunized twice at 4-week intervals with the inactivated recombinant rabies virus-vectored MERS-CoV vaccine, and sera were collected before the first immunization and at 4 and 8 weeks after the first immunization (**Figure 5A**). The antibody immune response against MERS-CoV as well as RABV was evaluated. As expected, sera of the rabies virus-vectored MERS-CoV vaccine immunized camels demonstrated strong neutralizing activity against both pseudotyped MERS-CoV derived from human and dromedary camel MERS-CoV spike sequences (**Figure 5B**). Satisfyingly, the immune sera from immunized camels at week 8 showed broad cross-neutralizing activity against multiple authentic viruses including the three major MERS-CoV clades (A, B, and C) (**Figure 5C**). Then the immune sera collected at 8 w.p.i were selected for the subsequent evaluation of prophylactic and therapeutic efficacies against live MERS-CoV (EMC/2012 strain) challenge in Ad5-hDPP4-transduced mice. Mice intravenously received 200 μl of immune sera 1 day before or after challenge; passive transfer of immune sera both resulted in a significant reduction of lung virus titers in both the prophylactic and therapeutic groups, indicating that the antibody responses elicited by the rabies virus-vectored MERS-CoV vaccine in camels are protective against MERS-CoV infection (**Figure 5D**). Besides, potent RABV-specific neutralizing antibodies far above 0.5 IU/ml were detected in sera of all camels at 8 weeks, with no significant differences being observed between sera harvested from the inactivated recombinant rabies virus-vectored MERS-CoV vaccine and the inactivated parental rabies virus vaccine immunized camels (**Figure 5E**).

### Protective Antibody Responses Elicited by the Rabies Virus-Vectored MERS-CoV Vaccine in Alpacas

**Figure 6:**
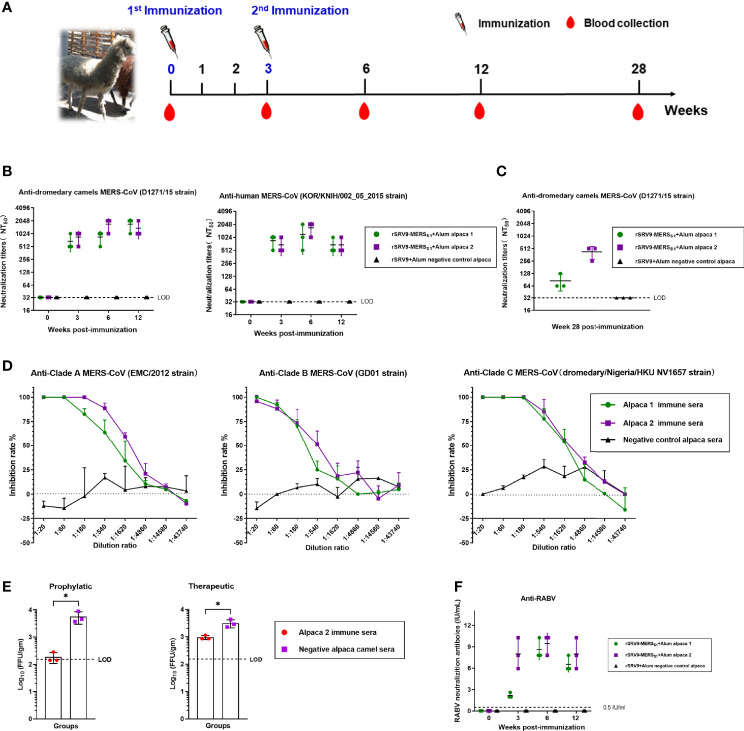
Humoral immune responses elicited by the inactivated rabies virus-vectored MERS-CoV vaccine in alpacas. **(A)** The schematic of the alpaca immunization experiment. Three alpacas received 3 ml of inactivated recombinant rabies virus-vectored MERS-CoV vaccine (n = 2) or phosphate-buffered saline (PBS) (n = 1) by multiple sites of subcutaneous (s.c.) injection in the neck two times at 3-week intervals. Sera of alpacas from each group were harvested before the first immunization and collected at 3, 6, and 12 weeks after the first immunization. **(B)** Sera cross-neutralizing activity against representative human and camel MERS-CoV strains (human MERS-CoV KOR/HIN strain and dromedary camel MERS-CoV D1271 strain) were evaluated by a pseudovirus-based neutralization assay. **(C)** Sera MERS-CoV-specific neutralizing antibodies measured 28 weeks after the first immunization by a pseudovirus-based neutralization assay. **(D)** The cross-neutralizing activity against live viruses among the known MERS-CoV clade A (EMC/2012 strain), clade B (ChinaGD01 strain), and clade C (dromedary/Nigeria/HKU NV1657 strain) were measured by focus reduction neutralizing assay. **(E)** Prophylactic and therapeutic efficacies of passive transfer with the rabies virus-vectored MERS-CoV vaccine alpaca immune sera collected at 6 w.p.i in MERS-CoV-infected Ad5-hDPP4-transduced mice. A total of 200 μl of alpaca immune sera collected were intravenously transferred 1 day before or after MERS-CoV (EMC/2012 strain) infection. Mice in the control group intravenously received the same dose of negative sera from healthy alpacas at the same time points. **(F)** Sera RABV-specific neutralizing antibodies were evaluated using fluorescent antibody virus neutralization (FAVN). Data are shown as mean ± SD. Data were obtained using GraphPad Prism version 9.0 (GraphPad software). An unpaired Student’s t-test (two-tailed) was used for statistical analysis, and the relevant p-values are indicated (not indicated in graph; *p < 0.05,).

Alpacas were immunized twice at 3-week intervals with the inactivated recombinant rabies virus-vectored MERS-CoV vaccine, and sera were collected at the indicated time for the detection of antibody responses against MERS-CoV as well as RABV (**Figure 6A**). As shown in **Figure 6B**, potent neutralizing antibody responses were elicited, and immunized alpaca sera neutralized both human and camel MERS-CoV pseudoviruses. Of note, immunized sera containing MERS-CoV-specific neutralizing antibodies remain robust and protective against MERS-CoV for at least 28 weeks after the first immunization (**Figure 6C**). So far, three clades (A, B, and C) of MERS-CoV were recognized; immunized alpaca sera demonstrated high neutralizing activities against multiple authentic MERS-CoV, including clade A, B, and C MERS-CoV (**Figure 6D**). Immune sera from immunized alpacas at week 6 were intravenously transferred into Ad5-hDPP4-transduced mice 1 day before or after the MERS-CoV challenge. Both mice from the prophylactic and therapeutic groups showed accelerated virus clearance in the lungs (**Figure 6E**), and immunized sera treatment showed a stronger protective effect in the prophylactic group, with reduced viral loads of approximately 1.5 logs in the lungs at 3 dpi. In addition, single-dose immunization of the rabies virus-vectored MERS-CoV vaccine induced potent anti-RABV neutralizing antibodies (above 0.5 IU/ml) in alpacas, which were higher than the standard 0.5-IU level considered protective by the WHO and World Organisation for Animal Health (OIE) (**Figure 6F**).

### MERS-CoV-Specific Alpaca VHHs Showed Potent Protective Effect Against MERS-CoV *In Vitro* and *In Vivo*

**Figure 7:**
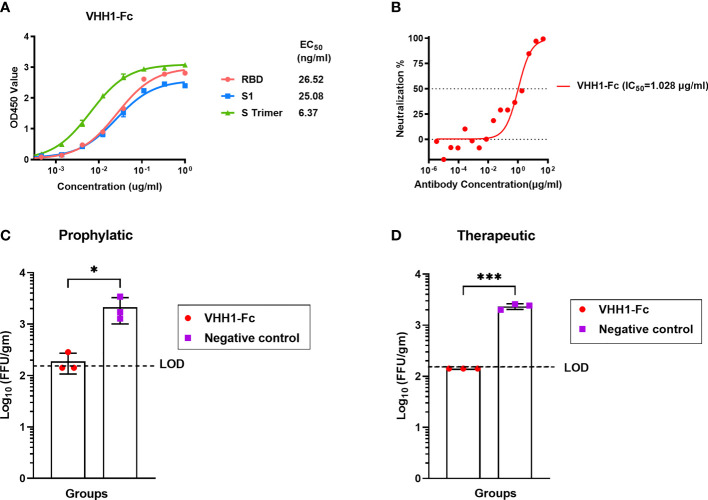
MERS-CoV-specific alpaca VHHs protected mice from MERS-CoV infection. **(A)** Binding activity of VHH1-Fc with MERS-CoV RBD, S1, and S trimer protein were measured by indirect ELISA. **(B)** Neutralizing activity against MERS-CoV prototype strain EMC/2012 was evaluated by pseudovirus neutralization assay. **(C)** Prophylactic and **(D)** therapeutic administration of VHH1-Fc protected MERS-CoV-infected mice (n = 3 per group). The prophylactic and therapeutic efficacies of VHH1-Fc were evaluated *in vivo* using Ad5-hDPP4-transduced mice challenged with MERS-CoV. VHH1-Fc was intravenously transferred 1 day before or after MERS-CoV (EMC/2012 strain) infection. The lungs were harvested at 3 days post-infection for viral titer determination. Mice in the control group (n = 3) were negative control antibodies (anti-HIV antibody 2G12) at the same time points. Data are shown as mean ± SD. Data were obtained using GraphPad Prism version 9.0 (GraphPad software). An unpaired Student’s t-test (two-tailed) was used for statistical analysis, and the relevant p-values are indicated (not indicated in graph; *p < 0.05, ***p < 0.001).

Two immunized alpacas were boosted with the inactivated recombinant rabies virus-vectored MERS-CoV vaccine three times; after boost immunization, MERS-CoV-specific alpaca VHHs were isolated for further characterization of the antibody response induced by the inactivated recombinant rabies virus-vectored MERS-CoV vaccine. Here, the alpaca immune VHH library displayed on the surface of yeast *S. cerevisiae* was constructed. Distinct VHH sequences were identified using MERS S trimer as a selection bait. To extend the *in vivo* half-life, the recombinant VHHs human-Fc-fused version (VHH-Fc) were constructed with a C-terminal human IgG1 Fc tag. One representative VHH-Fc was subsequently used for evaluation. VHH1-Fc can efficiently bound to MERS-CoV RBD, S1, and S trimer (EC_50_ value of half-maximal effective concentration, 26.52 ng/ml for RBD, 25.08 ng/ml for S1, and 6.37 ng/ml for S trimer) (**Figure 7A**). Neutralizing activity was assessed using MERS-CoV spike pseudotyped virus neutralization assay. VHH1-Fc demonstrated strong neutralizing activity against MERS-CoV (value of half maximal inhibitory concentration, IC_50_, 1.028 μg/ml) (**Figure 7B**). The prophylactic and therapeutic efficacies of VHH1-Fc were evaluated *in vivo* using Ad5-hDPP4-transduced mice challenged with MERS-CoV. As shown in **Figures 7C, D**, mice in both the prophylactic and therapeutic groups showed significantly reduced lung viral titer after infection as compared to control mice, and lung virus titer decreased 1 log at 3 dpi. Altogether, VHH1-Fc showed a potent protective effect against MERS-CoV *in vitro* and *in vivo*.

## Discussion

Novel coronaviruses emerge periodically in different areas globally. To date, three highly pathogenic beta-CoVs have been associated with zoonotic outbreaks, including SARS-CoV in 2002, MERS-CoV in 2012, and SARS-CoV-2 in 2019. Among these beta-CoVs, MERS-CoV infection has the highest case fatality rate (~34%) in patients, and infections range from asymptomatic or mild respiratory symptoms to severe acute respiratory disease and even death. As there is no licensed vaccine or specific treatment currently available, treatment is supportive for MERS patients (39). Till now, MERS-CoV continues to circulate, especially in the Arabian Peninsula (40). MERS-CoV is important in its own right but also as an example of a coronavirus that is highly pathogenic to humans.

The origins of MERS-CoV are not fully understood yet, but it is believed that it may have originated in bats and transmitted to camels at some time in the past (39, 41–43). So far, MERS-CoV has been identified in dromedaries in the Middle East, South Asia, and Africa, and multiple cases of animal-to-animal transmission among camelids (dromedary camels, llamas, and alpacas) as well as camel-to-human spillover have been reported (44–48). Although infected camelids show minor clinical signs from mild to moderate nasal discharge, the veterinary vaccine was highlighted in WHO Target Product Profiles for MERS-CoV Vaccines, which is noted that an animal vaccine strategy may be the best way to prevent human outbreaks and may have the faster development and licensing pathway (13).

In recent years, the rabies virus has been successfully used as a vaccine vector for many zoonotic diseases, including Ebola virus (EBOV) disease and COVID-19. Compared with another viral vector, the rabies vaccine vector has an excellent safety profile and impressive immunogenicity profiles in animals and humans (24–28). Previous studies by us and other research groups have demonstrated that the rabies vector could be desirable for the development of the MERS vaccine. Wirblich *et al*. found that an inactivated RABV/MERS-S-based vaccine could induce potent immune responses against MERS-CoV and RABV in mice (27). It was the first report of the RABV-vectored MERS-CoV vaccine, putting a tentative idea that this novel vaccine may be useful to protect target animals like camels, as well as humans. In another study, our team found that the rabies virus vector-based vaccine could induce remarkably earlier antibody response and higher levels of cellular immunity compared with the gram-positive enhancer matrix (GEM) particle vector, showing encouraging results of the use of rabies vector (49). Except for the replication-competent rabies vector, a replication-incompetent P-gene-deficient rabies vector was also used. Kato *et al*. generated an attenuated bivalent-vaccine against MERS and rabies, RVΔP-MERS/S1, and evaluated its humoral immunogenicity in mice after intraperitoneal inoculation. RVΔP-MERS/S1 induced significantly high titer neutralizing antibodies against RABV, while the neutralizing activity against MERS-CoV was relatively low, which required further animal-challenge tests to evaluate whether protective immunity was induced against MERS-CoV (50).

For vector-based vaccines, the presence of preexisting immunity is a major concern. Some of the commonly employed vectors, such as adenovirus especially adenovirus serotype 5 (Ad5), have a high prevalence of preexisting immunity in the host, which could significantly interfere with the subsequent immune response (51). However, this is not a problem for the rabies virus-vectored vaccines. Previous studies have shown that preexisting rabies immunity does not affect the immune response of the vaccine. Shuai *et al*. have proven that dogs previously vaccinated with annual rabies vaccine still developed increasing RABV and EBOV-specific responses after vaccination with the inactivated rabies virus-vectored EBOV vaccine, and a booster response could be induced in dogs both with and without previous experience of rabies vaccine (38). Similar to the rabies virus-vectored EBOV vaccine, our data show that significantly enhancing immune responses could be induced by the inactivated rabies virus-vectored MERS vaccine after boost immunization in both mice and camelids.

Notably, it is proved that transcription attenuation occurs in the process of RABV genome transcription. When rescuing recombinant virus based on RABV vector, the length and form of foreign antigen expression frame as well as its location in the RABV genome would be considered, as these factors have an effect on the expression of target antigen protein and the efficiency of rescued recombinant virus and its virus titer (34). Several studies have demonstrated that the length of inserted exogenous expression frame within 2,000 bp would be ideal (24–29). Besides, of the two subunits of MERS-CoV spike protein, S1 is the one that contains the RBD and the N-terminal, which are the regions against which more neutralizing antibodies are generated (52). Hence, in the current study, the S1 subunit rather than the full-length MERS-CoV spike protein was chosen and inserted between phosphoprotein and matrix protein genes of RABV. Recombinant rabies virus rSRV9-MERS_S1_ was successfully rescued with S1 protein of MERS-CoV incorporated into the virus particles, which provided the basis for the development of inactivated vaccine candidate. The vaccine form (live and inactivated) and delivery doses (one dose assayed for both live and inactivated vaccines and two doses assayed for inactivated vaccines) of the recombinant rabies virus-vectored MERS-CoV vaccine candidates were evaluated in mice, and the inactivated form with two immunizations was finally selected for further experiments in C57BL/6 mice, alpacas, and camels. Our results show that the rabies virus-vectored MERS-CoV vaccine induced robust and durable neutralizing antibodies and T-cell responses in mice. Further, the vaccination reduced viral replication and accelerated virus clearance in the lungs of MERS-CoV-infected Ad5-hDPP4-transduced mice. Protective antibody responses were also elicited by the rabies virus-vectored MERS-CoV vaccine in camels and alpacas. Of note, the MERS-CoV immunogen used for the vaccine construction was based on a consensus S glycoprotein; satisfyingly, the immune sera from immunized camelids showed broad cross-neutralizing activity against live viruses among the three major MERS-CoV clades (A, B, and C). For further characterizing the antibody response induced by the inactivated recombinant rabies virus-vectored MERS-CoV vaccine in camelids, MERS-CoV-specific variable domains of heavy-chain-only antibody (VHH) genes were also obtained from immunized alpacas and constructed with human-Fc-fused version, one of which showed potent prophylactic and therapeutic efficacies in an Ad5-hDPP4-transduced mouse model. Due to our accessible BSL-3 conditions, the *in vivo* protection evaluation in camelid models was lacking in this study. Future experiments should involve efficacy testing in alpacas or camels. Collectively, the current results demonstrate that the inactivated rabies virus-vectored MERS-CoV vaccine is safe, efficacious, and able to induce robust protective immune responses, representing a promising MERS camelid vaccine candidate and warranting further efficacy study in camelids of the main epidemic area, the Middle East area and Africa.

## Data Availability Statement

The raw data supporting the conclusions of this article will be made available by the authors, without undue reservation.

## Ethics Statement

The animal study was reviewed and approved by The Animal Welfare and Ethics Committee of the Changchun Veterinary Research Institute at the Chinese Academy of Agricultural Sciences and The Institutional Animal Care and Use Committees of the Guangzhou Medical University.

## Author Contributions

JZ, YZ, SY, and XX conceived and supervised the study. HC constructed and developed the rabies virus-vectored MERS-coronavirus vaccine. HC, EL, and XWW carried out all immunogenicity assays in mice, camels, and alpacas. YW, AZ, JS, ZZ, and LZ performed the MERS-CoV live virus neutralization assay and challenge experiments. JY and HC performed the selection and expression of recombinant MERS-CoV-S-specific VHHs. MH designed the consensus MERS-CoV S gene. HJW assisted in camels and alpaca immunization. HLW, HJ, NF, and YG provided advice on study design. HC, XWW, and YW carried out the data analysis. HC and XWW cowrote the paper, with contributions from QH, ZW, and XYW. All authors reviewed the manuscript.

## Funding

This work was supported by the National Natural Science Foundation of China (Grant No. 31902306 to HC, 81772191 and 82025001 to JZ, and 32000658 to LZ), Open Project of State Key Laboratory of Respiratory Disease (Grant No. SKLRD-OP-202009) to HC and YW, and Guangdong Science and Technology Foundation (Grant No. 2020A0505100063) to JZ.

## Conflict of Interest

The authors declare that the research was conducted in the absence of any commercial or financial relationships that could be construed as a potential conflict of interest.

## Publisher’s Note

All claims expressed in this article are solely those of the authors and do not necessarily represent those of their affiliated organizations, or those of the publisher, the editors and the reviewers. Any product that may be evaluated in this article, or claim that may be made by its manufacturer, is not guaranteed or endorsed by the publisher.

## Supplementary Material

Supplementary Figure 1 The generation of the consensus sequence of MERS-CoV S gene. A total of 194 sequences covering the known MERS-CoV clades were obtained from GenBank sequence database. And the consensus sequence of S gene was generated by choosing the most common amino acid at each position.
