# Supplementary figures and images for: Inactivated Rabies Virus Vectored MERS-Coronavirus Vaccine Induces Protective Immunity in Mice, Camels, and Alpacas
Source: Front Immunol. 2022 Jan 31;13:823949. doi: 10.3389/fimmu.2022.823949 (PMC8842186; doi:10.3389/fimmu.2022.823949)

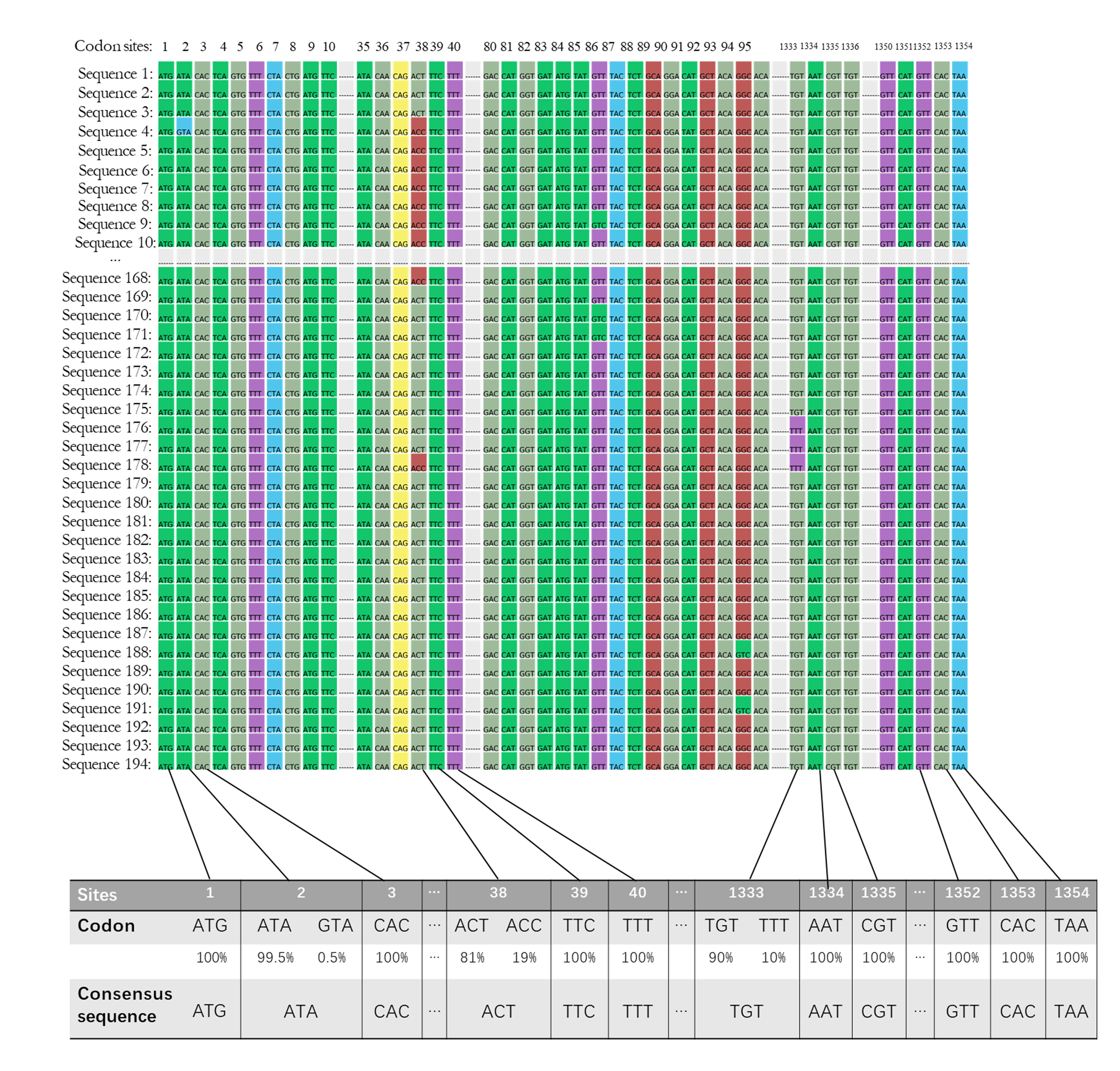

Supplement: Supplementary Figure 1 — The generation of the consensus sequence of MERS-CoV S gene. A total of 194 sequences covering the known MERS-CoV clades were obtained from GenBank sequence database. And the consensus sequence of S gene was generated by choosing the most common amino acid at each position. [file Image_1.tif]
